# A bibliometric analysis of perioperative rehabilitation research between 2005 and 2024

**DOI:** 10.3389/fresc.2025.1524303

**Published:** 2025-02-25

**Authors:** Juan Li, Fen Su, Qing Zhang, Guiqi Song

**Affiliations:** ^1^ School of Nursing, Anhui Medical University, Hefei, Anhui, China; ^2^ Education Section, The First Affiliated Hospital of USTC, Division of Life Sciences and Medicine, University of Science and Technology of China, Hefei, Anhui, China; ^3^ Department of Thoracic Surgery, The First Affiliated Hospital of USTC, Division of Life Sciences and Medicine, University of Science and Technology of China, Hefei, Anhui, China

**Keywords:** bibliometric analysis, rehabilitation, perioperative, CiteSpace, VOSviewer bibliometric analysis, VOSviewer

## Abstract

Effective rehabilitation can improve the prognosis of surgical patients, thereby enhancing their medical experience. In recent years, relatively more research is been carried out in this field; therefore, it is necessary to use bibliometric analysis to understand the development status and main research hotspots of perioperative rehabilitation, so as to determine the role of rehabilitation in the perioperative period. All documents related to perioperative rehabilitation and published from 2005 to 2024 were retrieved from the Web of Science Core Collection (Woscc). Number of articles, countries/regions, institutions, journals, authors, and keywords were analysed using VOSviewer and CiteSpace. A total of 829 studies on perioperative rehabilitation were included in the bibliometric analysis. The number of articles has steadily and rapidly increased since 2016. Over time, the publication outputs increased annually. There are 532 keyword nodes in total, of which the five keywords that appear most frequently are “surgery” “rehabilitation” “Outcome” “management” and “complications”. Research on the perioperative rehabilitation has developed rapidly. This study provides necessary information for researchers to understand the current status, collaborative networks, and main research hotspots in this field. In addition, our research findings provide a series of recommendations for future studies.

## Introduction

At least 300 million people worldwide undergo major surgeries every year. The success of surgery depends not only on exquisite surgery but also on postoperative rehabilitation and nursing (1, 2). Postoperative rehabilitation nursing can promote blood circulation, reduce tissue edema, improve muscle function, promote wound healing, prevent postoperative complications, and increase surgical success rate (3). Surgical rehabilitation refers to the process of helping patients recover their physical function and improve their quality of life through a series of rehabilitation measures after surgery (4). It mainly includes the following aspects: postoperative rest and recovery; pain management; functional exercise; dietary regulation; and psychological support (5, 6). All surgical patients require a rehabilitation process. Effective rehabilitation exercises can reduce the incidence of postoperative complications, shorten hospitalization time, lower medical costs, and improve patients' perceived health-related quality of life. Rehabilitation strategies are typically multidisciplinary (7). Rehabilitation training methods cover a wide range of methods, including, but not limited to, active or passive training, psychological rehabilitation, occupational therapy, cognitive rehabilitation, and cardiac rehabilitation (8, 9). In summary, rehabilitation plays an important role in perioperative management of patients undergoing various surgeries.

In recent years, many scholars have applied rehabilitation to various disciplines, but few reports have analyzed the development trends and hotspots of surgical rehabilitation. Bibliometrics is an interdisciplinary science that uses mathematical and statistical methods to quantitatively analyze various knowledge carriers (10–12). Bibliometric analysis is an important tool for researchers to grasp new trends in current scientific research. It is a recognised as systematic analysis technique and plays an important role in management decisions and clinical research (13). It can help researchers identify research priorities and trends on specific topics, and the results may be instructive for future research (13, 14). This study adopts bibliometric methods, where it utilises relevant literature from the Web of Science database, CiteSpace and VOSviewer software to extract and analyze research titles and abstracts of papers within the research scope. High frequency keywords are selected to quantify the current status and hotspot distribution of global rehabilitation research, providing a theoretical basis for relevant researchers.

## Methods

### Data collection

Web of Science Core Collection (Woscc) is an online database containing standardized and up-to-date reference datasets for scientific research and analysis, of which SCIEXPANDED is considered the most suitable database for bibliometric analysis (15). Based on the Wos database, the advanced search page takes “rehabilitation” and “perioperative” as the themes, and the time range is from January 1, 2005 to May 20, 2024. The relevant literature is retrieved, and the scientific and technological achievement literature that is not directly related to the theme is screened and eliminated. The literature type is selected as the paper or review paper. Finally, 829 articles were obtained from the wos database. Subsequently, the processed data were imported to CiteSpace version 6.1.r6 (16) and VOSviewer v.1.6.15.0 (17) for bibliometric analysis.

### Data analysis

Citespace visual analysis software was used to measure the obtained literatures in the field of rehabilitation and surgery research and draw relevant knowledge charts. VOSviewer was used to visually analyze the authors, research categories, countries, journals, keywords, and timelines and identify the frontier hotspots. Knowledge map is a series of different graphs that show the development process and structural relationship of knowledge. It uses visualization technology to describe knowledge resources and their carriers, mining, analyzing, constructing, drawing and displaying knowledge and the relationship between them. On the basis of the chart analysis, we read the literature deeply, and summarised the research status and hot spots of rehabilitation and surgery. The wos data acquisition process is as follows: First, four folders are created, which are composed of data Input, output and project naming. Secondly, open Wos, input the keywords “rehabilitation” and “perioperative” from the advanced search interface to search, check all the literature related to the subject and export it, click “other file formats” when exporting the literature, and select “plain text” format in the file format box to obtain the converted data, save in input folder. Open the cite space software and copy the converted data from the output folder to the data folder. Create a new project in the city space software, enter the task name in the “title” box, select the built project and data folders in the corresponding file option box, select “web of science”, and click “save”, that is, the project has been built. Then, in the function selection area, select the author, institution, keyword and other function options to be analyzed, and click the. The key categories of “author”, “institution”, and “country” are distributed in the node cooperation network to explore the cooperation in the research field. Carry out keyword co-occurrence analysis and clustering analysis on “keyword”, explore research hotspots and predict the development trend of this field in the future. Click “go” and “visualize” to get the visualization results.

## Results

### Publication trends

**Figure 1:**
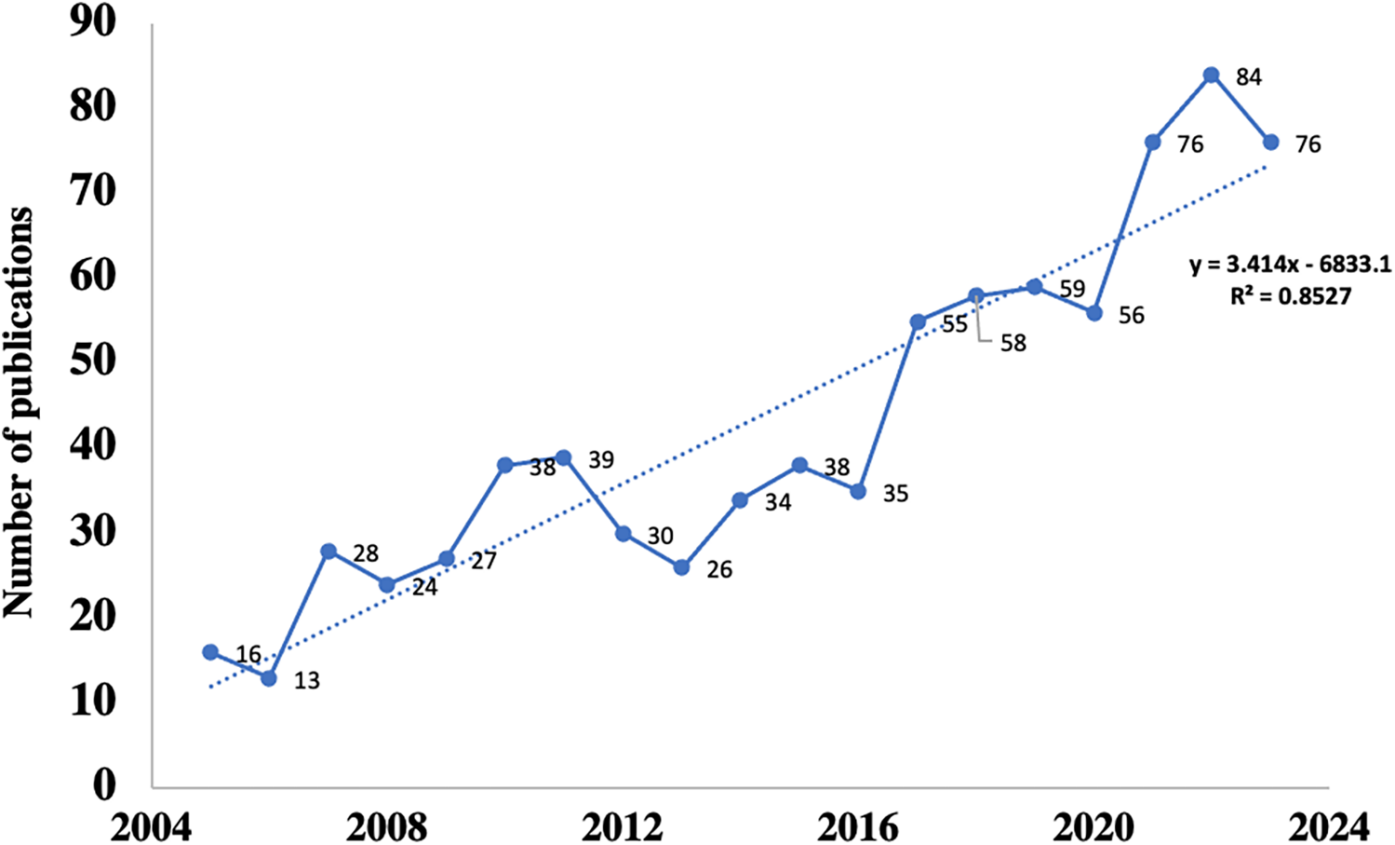
Annual publication trend of perioperative rehabilitation.

The distribution of articles in every period indicates the general examination patterns in the field. From Figure 1, it can be seen that the number of articles on perioperative rehabilitation has been increasing year by year. From 2006 to 2010, the publication volume gradually increased, while there was a decreasing trend between 2010 and 2012. In addition, the number of articles has steadily and rapidly increased since 2016, indicating that surgery and rehabilitation have received special attention during this period.

### Analysis of keywords and research frontiers

**Figure 2:**
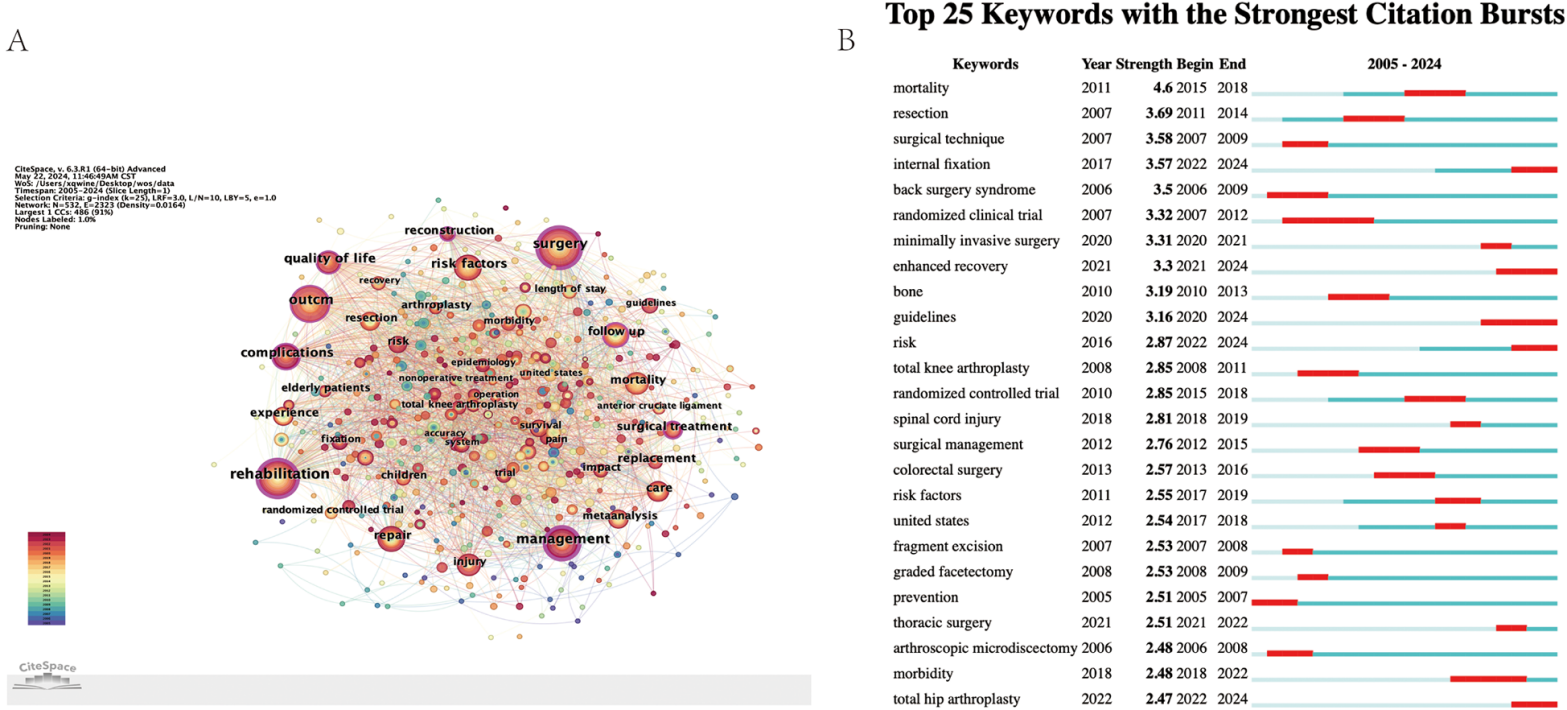
Key words analysis of perioperative rehabilitation related research publications. **(A)** Perioperative rehabilitation related keywords co-occurrence network view. **(B)** The top 25 keywords most frequently cited.

In Figure 2A, there are 532 keyword nodes in total, of which the five keywords that appear most frequently are “surgery” “rehabilitation” “Outcome” “management” and “complications”. Figure 2B shows the evolution of keywords from 2005 to 2024. The results show that the top 25 keywords with the largest number of citation bursts, among which “mortality” has the largest number of bursts (*n* = 4.6). The first five most important clusters represent the current status of perioperative rehabilitation, and surgical techniques remain the main research hotspots.

### Analysis of countries and institution

**Figure 3:**
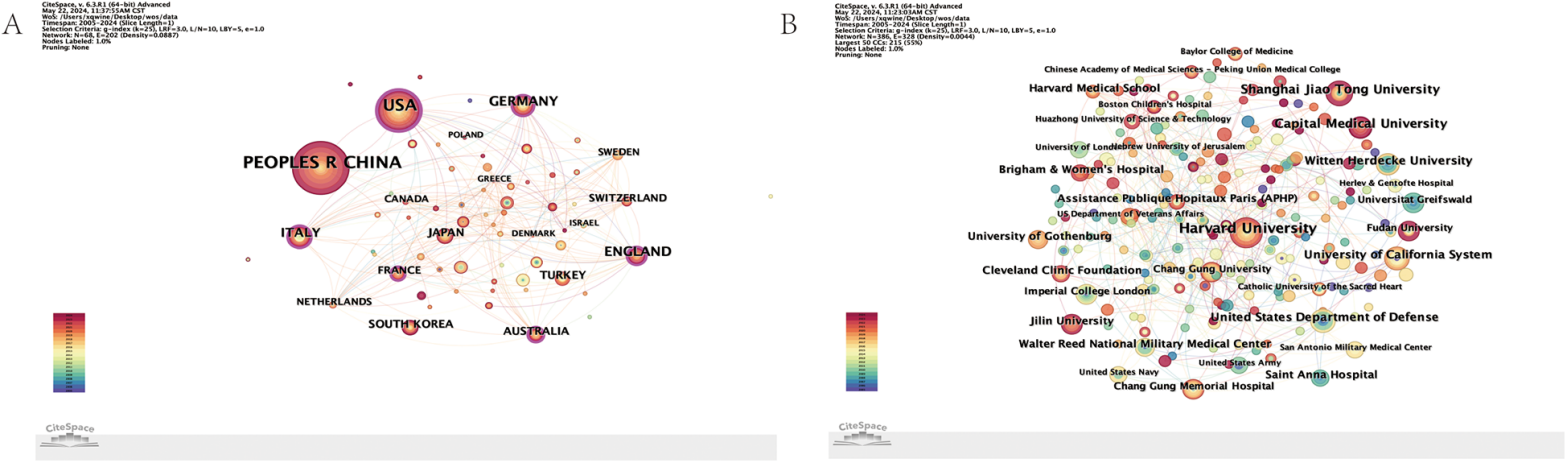
Countries and institutions with the most research on perioperative rehabilitation. **(A)** Number of publications in the top 20 countries citations/50 and average citations *10. **(B)** Perioperative rehabilitation related research network visualization map of cooperative institutions in various countries.

Figure 3 shows the corresponding cooperation network between the top ten most productive countries and institutions, with publications from China, the United States and Germany ranking in the top three respectively. Figure 3A shows the top ten countries with the most perioperative rehabilitation related research, and the United States has the most perioperative rehabilitation related publications. Figure 3B shows the interaction diagram among various institutions, with Harvard University publishing the most. In addition, the top ten institutions in terms of publishing frequency are shown in Table 1.

**Table 1:** Top 10 authors of publications.

Rank	Authors	Record Count	Affiliations	H-index
1	Ruetten, Sebastian	8	Saint Anna Hospital	23
2	Godolias, Georgios	8	Ruhr University Bochum	26
3	Komp, Martin	8	Ruhr University Bochum	18
4	Merk, Harry	8	Ruhr University Bochum	18
5	Buckenmaier, Chester C	2	Def & Vet Ctr Integrat Pain Management	24
6	Zhu, Yu	2	Beijing Normal University	1
7	Chen, Kaiwen	2	Hefei Normal University	1
8	Lyu, Feizhou	2	Fudan University	3
9	Jiang, Jianyuan	2	Guangxi Normal University	7
10	Xia, Xinlei	2	Nanchang University	18

### Analysis of authors

**Figure 4:**
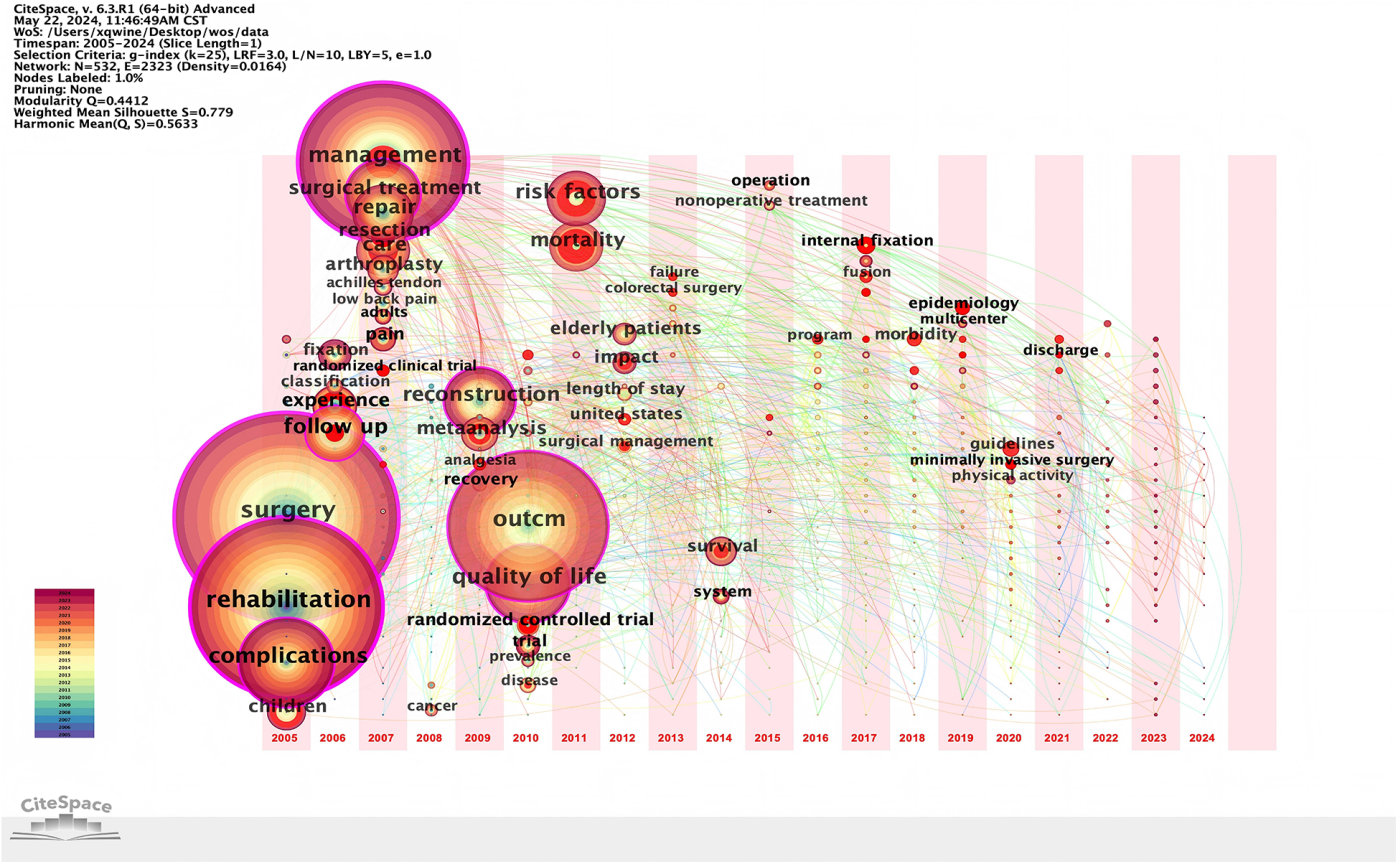
Visualization of perioperative rehabilitation related researcher cooperation network.

The top three authors of perioperative rehabilitation related literatures collected by Wos are “Ruetten Sebastian”, “Godolias Georgios”, “Komp Martin”, “Merk Harry”, The authors such as “Chen Kaiwen”, “Lyu Feizhou” and others have a high degree of cooperation (Figure 4). Co-cited authors are defined as at least two authors who have been cited in at least one subsequent paper. Among the top 10 co-cited authors, 4 have been cited more than 8 times, with Ruetten Sebastian, Godolias Georgios, Komp Martin and Merk Harry being cited the most (Table 2).

**Table 2:** Top 10 cited references of publications.

Rank	Title	Authors	Journal	Citations
1	Extending the CONSORT statement to randomized trials of nonpharmacologic treatment: explanation and elaboration	Boutron, I; Moher, D; Altman, DG; Schulz, KF; Ravaud, P	ANN INTERN MED	1,817
2	Full-endoscopic interlaminar and transforaminal lumbar discectomy vs. conventional microsurgical technique -: a prospective, randomized, controlled study	Ruetten, S; Komp, M; Merk, H; Godolias, G	SPINE	520
3	A protocol is not enough to implement an enhanced recovery programme for colorectal resection	Maessen, J; Dejong, CHC; Hausel, J; Nygren, J; Lassen, K; Andersen, J; Kessels, AGH; Revhaug, A; Kehlet, H; Ljungqvist, O; Fearon, KCH; von Meyenfeldt, MF	BRIT J SURG	369
4	Mandibular reconstruction using stereolithographic 3-dimensional printing modeling technology	Cohen, A; Laviv, A; Berman, P; Nashef, R; Abu-Tair, J	ORAL SURG ORAL MED O	263
5	Full-endoscopic cervical posterior foraminotomy for the operation of lateral disc herniations using 5.9-mm endoscopes - a prospective, randomized, controlled study	Ruetten, S; Komp, M; Merk, H; Godolias, G	SPINE	245
6	Recommendations for the management of patients after heart valve surgery	Butchart, EG; Gohlke-Bärwolf, C; Antunes, MJ; Tornos, P; De Caterina, R; Cormier, B; Prendergast, B; Iung, B; Bjornstad, H; Leport, C; Hall, RJC; Vahanian, A	EUR HEART J	245
7	Use of newly developed instruments and endoscopes: full-endoscopic resection of lumbar disc herniations via the interlaminar and lateral transforaminal approach	Ruetten, S; Komp, M; Merk, H; Godolias, G	J NEUROSURG-SPINE	218
8	A new full-endoscopic technique for the interlaminar operation of lumbar disc herniations using 6-mm endoscopes: prospective 2-year results of 331 patients	Ruetten, S; Komp, M; Godolias, G	MINIM INVAS NEUROSUR	200
9	Surgical treatment for lumbar lateral recess stenosis with the full-endoscopic interlaminar approach vs. conventional microsurgical technique: a prospective, randomized, controlled study	Ruetten, S; Komp, M; Merk, H; Godolias, G	J NEUROSURG-SPINE	195
10	Recurrent lumbar disc herniation after conventional discectomy a prospective, randomized study comparing full-endoscopic interlaminar and transforaminal versus microsurgical revision	Ruetten, S; Komp, M; Merk, H; Godolias, G	J SPINAL DISORD TECH	171

### Analysis of contributing journals

As shown in Table 3, the most frequently cited journal is ANN INTERN MED (1,817 times), indicating that this journal may have significant implications for surgery and rehabilitation, followed by SPINE (520 times). The influence of a journal depends on the number of joint citations. The most cited article among them is the one titled “Extending the CONSORT (Consolidated Standards of Reporting Trials) Statement to Randomized Trials of Nonpharmacologic Treatment: Explanation and Elaboration” published by Boutron et al, and the IF (2023) of journal “ANNALS OF INTERNAL MEDICINE” is 19.8. In this article, By expanding CONSORT to help improve the reporting of non pharmacological treatments in RCTs, the CONSORT statement is a 22 item checklist and flowchart that addresses the issue by improving the reporting of randomized controlled trials. However, there is ample evidence to suggest that the reporting of non pharmacological trials still needs improvement, so the CONSORT team has expanded the statement (18). The second most cited article was “Full-endoscopic interlaminar and transforaminal lumbar discectomy vs. conventional microsurgical technique: a prospective, randomized, controlled study”. Through a 2-year follow-up of 178 patients undergoing total endoscopic or microsurgery, a randomized controlled study was conducted to compare the effectiveness of total endoscopic interbody surgery with conventional microsurgical techniques for lumbar discectomy. The results showed that the clinical efficacy of total endoscopic technology was comparable to that of microsurgical technology, while also having advantages in surgical techniques and reducing trauma (19).

**Table 3:** Top 10 institutions by frequency of publications.

Rank	Frequency	Institutions	Centrality	Degree
1	18	Harvard University	0.04	21
2	10	Shanghai Jiao Tong University	0	1
3	10	Capital Medical University	0	1
4	10	Sichuan University	0	0
5	9	University of California System	0.02	10
6	9	United States Department of Defense	0	7
7	9	Witten Herdecke University	0	3
8	8	Assistance Publique Hopitaux Paris (APHP)	0.03	23
9	8	Cleveland Clinic Foundation	0.02	8
10	8	Jilin University	0	2

### Cluster analysis

**Figure 5:**
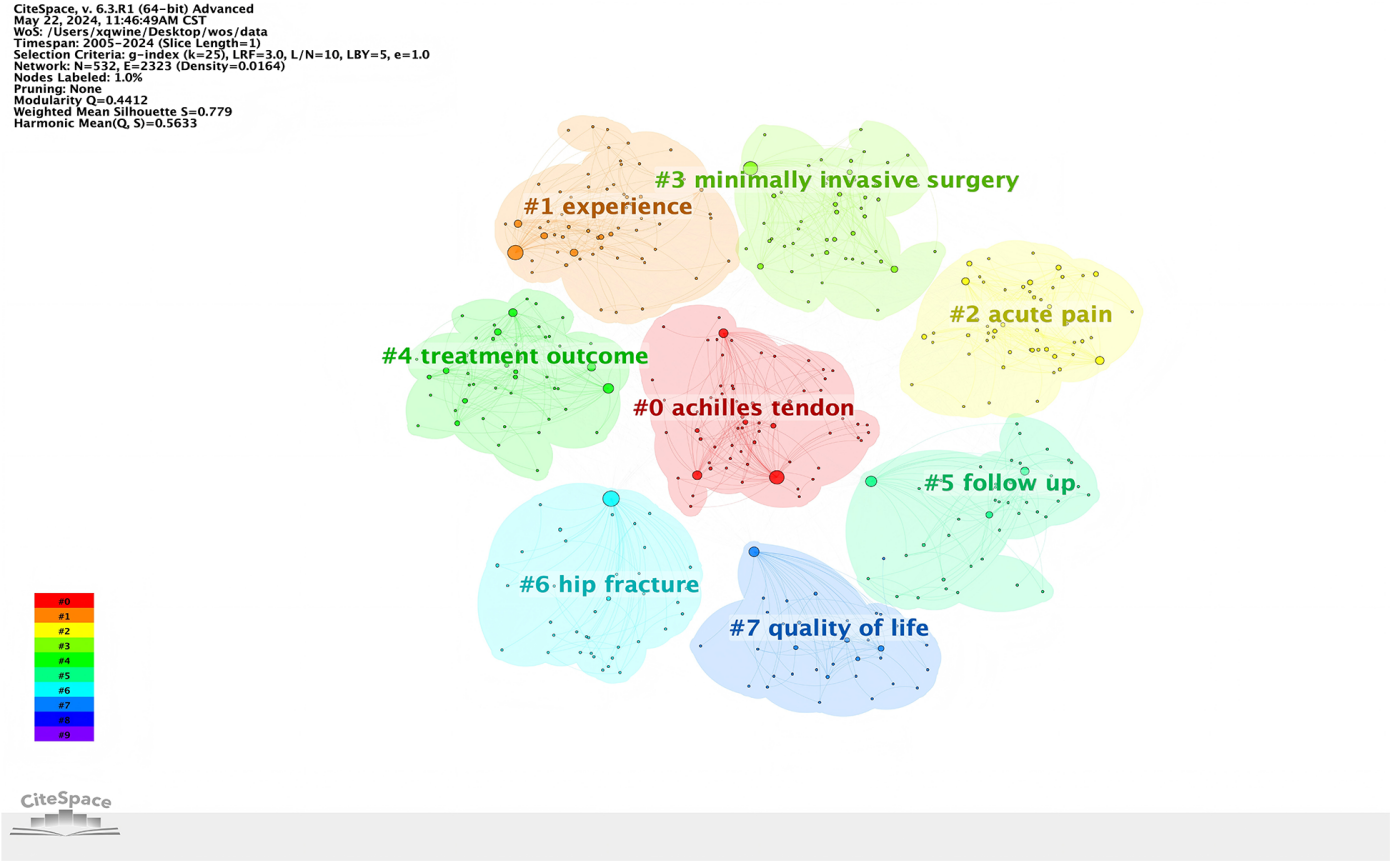
Keywords clustering map of perioperative rehabilitation.

Based on the co-occurrence of keywords, the local linear regression algorithm was used to cluster the keywords and generate the keyword clustering map. The number of literature studies on Achilles tendon, experience, acute pain and minimally invasive otology was the largest, and the research heat was the highest (Figure 5).

### Keywords timeline and time zone analysis

**Figure 6:**
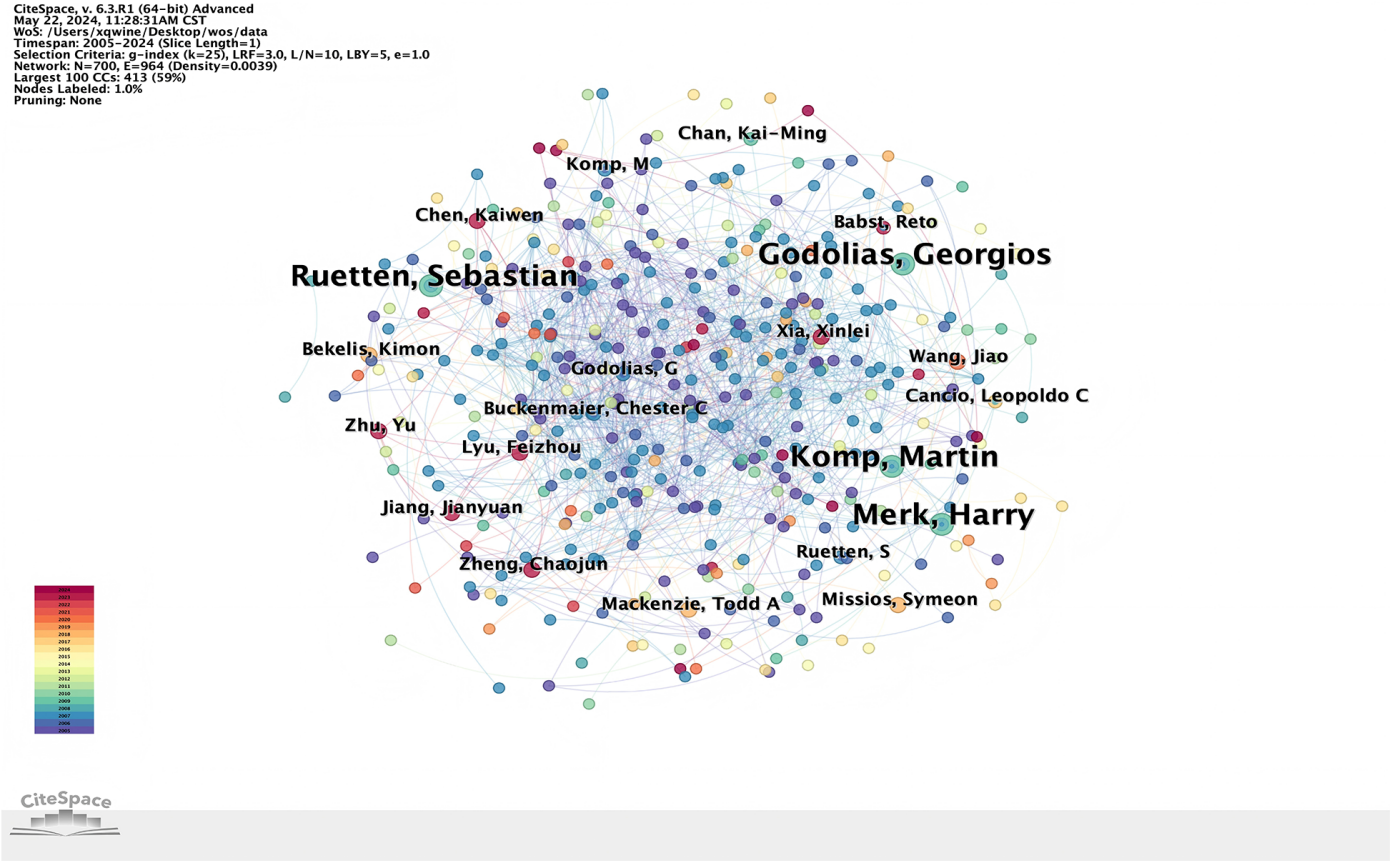
Timeline of perioperative rehabilitation.

**Figure 7:**
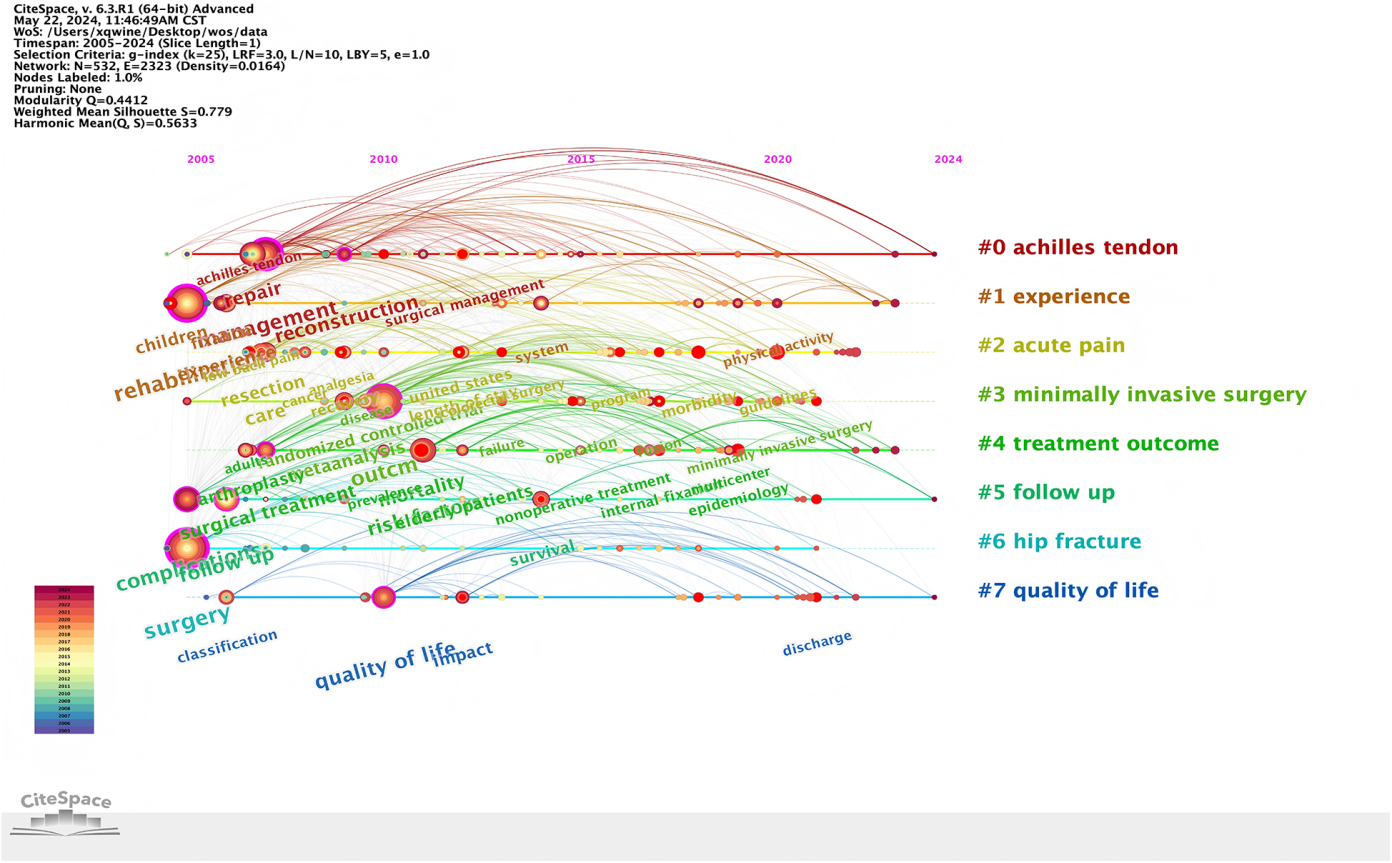
Time zone map of perioperative rehabilitation.

The keyword timeline graph can show the changes of keywords from the time dimension, and the length of the horizontal line corresponding to each cluster represents the time span of the cluster. As shown in Figure 6, the Timeline view of keywords shows the changes of high-frequency keywords over time. As the search phrases of this study, “children” and “complications” first appeared at the beginning of the temporal evolution of clustering. In the initial stage of development, cluster “Achilles tendon”, “experience” and “acute pain” initially surfaced. Most of the remaining clusters are formed in the stage of slow development. Run CiteSpace 6.1.r6 to select timezone view on the basis of keyword co-occurrence to generate a keyword time zone map based on the time zone view layout. As shown in Figure 7, keywords such as “discharge” “guidelines” “minimally invasive surgery” began to appear in the past three years.

## Discussion

This study conducted a bibliometric analysis of perioperative and rehabilitation from 2004 to 2024, From Figure 1, it can be seen that the number of articles on perioperative rehabilitation has been increasing year by year. It is expected that research related to perioperative rehabilitation will continue to increase in the future.

ANN INTERN MED AND SPINE was cited the most. Among the top 10 journals, two have an impact factor exceeding 10 (EUR HEART J, ANN INTERN MED), three have an impact factor of 2–5 (SPINE, J NEUROSURG-SPINE, NEUROSUR) and one has an impact factor of 5–10 (BRIT J SURG). These results indicate that the research quality of perioperative rehabilitation is relatively high, with China contributing the largest number of papers published in the aforementioned journals, followed closely by the United States and Germany. In terms of traditional institutions, the largest clusters are labeled as “achilles tendons”, “experience”, and “acute pain”. Achilles tendon rupture is a common injury, and surgical repair carries a certain risk of infection. According to reports, the infection rate after surgical repair ranges from 0.2% to 3.6%. However, exercise based rehabilitation is internationally recognized as an effective treatment method that can improve quality of life and reduce the risk of readmission for patients (20). Exercise-based cardiac rehabilitation is recognised internationally as an effective therapy to improve quality of life and reduce the risk of hospital readmission for individuals diagnosed with acute coronary syndrome (21). In addition, studies have shown that post-stroke patients mainly receive rehabilitation treatment based on certain experience, and there seems to be no single goal setting method in stroke rehabilitation (22). Acute pain typically has a recognizable temporal and causal relationship with injury or disease, and is typically defined as pain that has recently occurred and may have a limited duration (23). In clinical practice, acute pain refers to pain that lasts for less than 3 months. At present, there are two main types of methods for treating acute pain, namely drug therapy and non drug therapy (24). The etiology of acute pain is multifactorial, and surgical procedures can cause damage to tissues. Surgical damage triggers countless reactions in the pain matrix, and preventing and reducing postoperative pain is the core responsibility of medical staff, which is extremely important in surgical rehabilitation (25).

Keyword clustering analysis found that among the top 10 keywords, in addition to keywords related to search terms, research in this field mainly focuses on mortality rate, surgical resection, and internal fixation. Mortality rate is one of the most important outcomes of surgery, and without risk stratification, it is difficult to explain the intraoperative mortality rate itself (26). Accurate risk stratification tools are the key to intraoperative diagnosis/treatment approaches. Nearly 300 million surgical procedures are performed globally every year, and basic surgical care can prevent at least 77.2 million cases of disability-adjusted life-years (DALYs) (27). Surgical resection is currently one of the main methods to treat cancer related diseases, mainly including pancreatic cancer (28), bone tumor (29), colorectal cancer (30), carotid aneurysm (31), etc. Internal fixation has become a pillar of the surgical profession, and the proximal femur is an important load-bearing area and a common site of benign lesions during surgery. Vigorous postoperative rehabilitation is associated with a lower incidence of deep venous thrombosis which is a very significant problem especially in Caucasian populations because of their predisposition to forming venous clots with immobilization (32). Preventive fixation is considered to cause less damage than pathological fractures (33).

One of the most important factors in evaluating the quality of research on perioperative and rehabilitation is the number of citations, which helps determine the areas of interest in the study. Through reading and analyzing the top 10 frequently cited literature, the results showed that full endoscopy, three-dimensional printing modeling technology, non pharmacological treatment randomized trials, etc. were widely studied by researchers in the field at specific times (Table 3). The following are the most cited papers, highlighting various research hotspots related to the rehabilitation of surgical patients. One of the research reports indicates that total endoscopic surgery is a sufficient and safe supplement and alternative to microsurgery, and has advantages in surgical techniques and reducing trauma (19). In traditional perioperative care, the length of hospital stay after colorectal surgery is usually 1–2 weeks. Then, by identifying factors that delay postoperative recovery and combining a series of intervention measures, surgical stress and dysfunction are reduced, and rehabilitation is strengthened. Multimodal rehabilitation can reduce hospitalization time and costs, accumulating experience for postoperative recovery of colorectal cancer patients (34). In highly cited literature, the main research direction is closely related to perioperative rehabilitation content, among which the application of total endoscopy technology in orthopedics is the most popular. In summary, current research related to perioperative rehabilitation mainly focuses on “management” “surgery” “rehabilitation” “complications” “outcome” “quality of life” and so on. However, in recent years, research on the survival rate of surgical patients has also been involved, and there has been an improvement in rehabilitation related experience, laying the foundation for further strengthening patient rehabilitation.

This is our first study to use CiteSpace's co-occurrence and citation method for bibliometric analysis and visualization of rehabilitation and perioperative. However, this study heavily relies on public databases and does not cover other commercial databases such as Scopus, Medline, and CNKI. Therefore, the data may not be comprehensive, but this is also a limitation caused by databases and software. WOS has the characteristics of wide coverage and strong authority, so our research content is reliable.

## Data Availability

The raw data supporting the conclusions of this article will be made available by the authors, without undue reservation.
